# Effect of 20 wt% Glass Fiber Reinforcement on the Mechanical Properties and Microstructure of Injection-Molded PA6 and PA66

**DOI:** 10.3390/polym18030357

**Published:** 2026-01-29

**Authors:** Serhad Dilber, Lütfiye Dahil

**Affiliations:** Mechanical Engineering Department, Istanbul Aydin University, Beşyol Mah. Inönü Cad. No:38, Istanbul 34295, Turkey; serhaddilber@stu.aydin.edu.tr

**Keywords:** polyamide (PA6; PA66), glass fiber reinforcement, injection molding, mechanical properties, SEM analysis, finite element analysis

## Abstract

This study investigates the mechanical performance and surface morphology of polyamide-based materials commonly used in plastic injection molding. Two resins, PA6 and PA66, were analyzed in both neat and 20 wt% glass fiber-reinforced (GF20) forms. The influence of reinforcement and material type on tensile strength and ductility was examined through integrated experimental and numerical approaches, complemented by microstructural and elemental analyses. PA6 and PA66 specimens were produced in accordance with ISO 527, and tensile tests revealed a significant increase in elastic modulus and tensile strength with glass fiber reinforcement, accompanied by a reduction in elongation at break. Flammability was evaluated via Glow Wire and Tracking tests. SEM–EDS analyses provided insights into fracture morphology and elemental distribution, showing that fiber–matrix interfacial debonding and fiber pull-out dominated failure in reinforced specimens, whereas neat polymers exhibited homogeneous surfaces. Finite element simulations performed in ANSYS Explicit Dynamics supported the experimental findings by identifying stress concentration zones and failure initiation regions. Although numerical simulations successfully captured stress distribution trends, quantitative differences were attributed to idealized modeling assumptions and processing-induced microstructural effects. Overall, this work provides a comprehensive assessment of the reinforcement effects in PA6 and PA66 systems, offering valuable guidance for material selection and design optimization in polymer-based engineering components.

## 1. Introduction

Polymer-based materials have become indispensable in modern engineering applications owing to their low density, high design flexibility, cost efficiency, and excellent suitability for large-scale manufacturing, particularly in weight-sensitive and high-performance industrial sectors [1]. Plastic injection molding is one of the most widely used techniques for processing polyamide composites, as it allows efficient fiber impregnation and desirable part geometry with high reproducibility [2]. However, pure polyamides such as PA6 and PA66 exhibit limitations including moisture absorption, dimensional instability, and restricted mechanical strength under demanding conditions [3,4].

Polyamide 6 (PA6) and Polyamide 66 (PA66) are widely used engineering thermoplastics owing to their high impact strength, good abrasion resistance, and favorable cost–performance balance, which enable their application in load-bearing and structural components across various industrial sectors [5,6]. Nevertheless, efforts to enhance their mechanical and thermal performance have led to the widespread use of glass fiber reinforcement, which is generally reported to improve tensile strength, flexural modulus, dimensional stability, wear resistance, and heat deflection temperature, particularly under optimized processing conditions that ensure effective fiber orientation, sufficient fiber length retention, and strong fiber–matrix interfacial bonding [7,8,9,10,11]. Numerous studies confirm that 20–30 wt% glass fiber offers an optimal balance between stiffness enhancement and processability in polyamide matrices [12,13]. Recent studies have further emphasized that the mechanical performance of glass fiber-reinforced polyamides is highly sensitive to processing-induced fiber orientation, fiber length degradation, and interfacial integrity, particularly in injection-molded short glass fiber systems [14,15,16]. The mechanical behavior of these composites is therefore strongly governed by fiber–matrix adhesion, fiber orientation, and fiber length distribution, which directly control the effectiveness of stress transfer [17,18,19].

In injection-molded composites, molding parameters such as temperature, pressure, and injection speed play a decisive role in defining fiber orientation patterns, resulting in pronounced variations in mechanical performance [2,20]. Moreover, recent numerical and experimental studies have demonstrated that idealized simulation assumptions may lead to an overestimation of mechanical properties when processing-induced damage mechanisms—such as fiber breakage, orientation dispersion, and interfacial debonding—are not explicitly considered [7,14,16]. In parallel, extensive experimental investigations on PA6, PA66, PA6 GF30, and PA66 GF30 systems have focused on tensile behavior, fatigue performance, thermal dependence, morphological characteristics, and moisture-induced degradation [15,16,21,22,23]. Additional studies addressing hybrid reinforcements, PA6/PA610 blends, and manufacturing-induced morphology asymmetry further highlight the strong sensitivity of polyamide composites to microstructural features and processing history [24,25,26,27,28].

Although these studies provide valuable insights, no comprehensive research has simultaneously examined the mechanical, morphological, elemental (SEM–EDS), fire-resistance, and simulation-supported stress–strain characteristics of PA6 and PA66 materials reinforced with the same glass fiber content (20 wt%) under identical processing conditions. Literature also lacks a comparative understanding of how PA6 and PA66 matrices respond differently to equal reinforcement levels in terms of stiffness, elongation, fracture morphology, and numerical–experimental agreement.

Therefore, the present work aims to fill this gap by producing PA6, PA6 GF20, PA66, and PA66 GF20 materials through plastic injection molding and conducting tensile testing, SEM–EDS characterization, fire-resistance evaluation, and finite-element simulations to reveal the relationships between microstructure, interfacial bonding, stress distribution, and mechanical performance. This integrated approach provides valuable insights for the automotive, white goods, and electronics industries, where lightweight and thermally stable materials must offer reliable mechanical integrity and improved safety characteristics.

## 2. Materials and Methods

In this study, four types of polyamide-based materials were investigated: unreinforced PA6 and PA66 (natural), and glass fiber-reinforced PA6 GF20 and PA66 GF20. All materials were produced via plastic injection molding under processing conditions recommended by the manufacturers’ technical data sheets (TDS), ensuring consistent melt temperature, injection pressure, and cooling rate.

Tensile tests were conducted on the molded specimens to evaluate their mechanical behavior. The fracture surfaces of the tested samples were analyzed using Scanning Electron Microscopy (SEM) and elemental mapping to examine surface morphology and molecular composition. Additionally, the materials were subjected to Glow Wire and Tracking flammability tests to assess fire resistance, as well as thermal aging tests to determine their heat resistance under elevated temperature conditions.

### 2.1. Plastic Injection Sample Production

All specimens—unreinforced PA6 and PA66, as well as 20 wt% glass fiber–reinforced PA6 GF20 and PA66 GF20—were fabricated using a HAITIAN HTF90W (Ningbo, China) injection molding machine (90-ton clamping force) in accordance with the ISO 527-1 tensile specimen geometry [29]. Processing parameters were selected based on the manufacturers’ technical data sheets (TDS) to ensure homogeneous melt flow, prevent fiber degradation, and achieve dimensional stability. Prior to injection, all materials were dried at 80 °C for a minimum of 4 h to remove moisture and avoid hydrolytic degradation during processing. The injection molding parameters used for specimen production are summarized in Table 1.

Samples 1, 2, and 3 correspond to three independent specimens tested under identical conditions to ensure repeatability and statistical consistency. The injection molding temperature profiles differed between the PA6- and PA66-based systems. For PA6 and PA6 GF20, the barrel temperature was set in the range of 240–270 °C, while for PA66 and PA66 GF20, a temperature profile approximately 40 °C higher was applied to compensate for their higher melting point and viscosity. Mold temperature was maintained between 80 and 90 °C for all materials, and the injection pressure was controlled within 70–90 MPa. Cooling cycles were adjusted to eliminate surface defects and minimize shrinkage.

**Figure 1:**
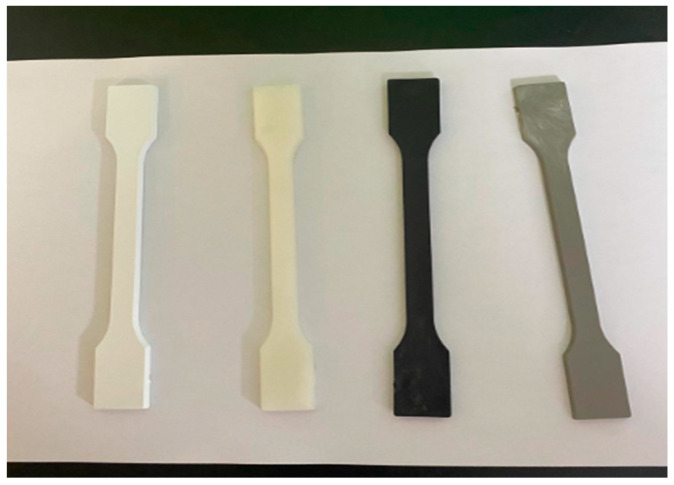
ISO 527-1 tensile test specimen geometry used for PA6, PA6 GF20, PA66, and PA66 GF20 materials.

All specimens were molded using the same cavity design, whereas processing parameters were modified only to meet the specific thermal requirements of each polymer type. The ISO 527-1 tensile test specimen geometry is shown schematically in Figure 1.

### 2.2. Tensile Test Methodology

Tensile tests were performed using a Zwick/Roell universal testing machine (Ulm, Germany) (model Xforce P, 20 kN capacity) in accordance with ISO 527-1 standards. All specimens were tested at room temperature under a constant crosshead speed of 50 mm/min. The applied load and corresponding elongation were continuously recorded through the machine’s integrated sensor and data acquisition system.

For each material group, three replicate tests were conducted to ensure statistical reliability. The resulting stress–strain curves were used to determine the elastic modulus (E), ultimate tensile strength (R_m_), and elongation at break (%At).

### 2.3. Flammability Test

Flame retardancy and surface tracking resistance of the materials were evaluated using Glow Wire and Tracking Index tests in accordance with IEC 60695-2-12 [30] and IEC 60112 standards [31], respectively. Tests were conducted at PTL Dr. Grabenhorst GmbH (Crailsheim, Germany) using certified equipment. The glow wire test was performed to determine the ignition temperature and self-extinguishing behavior of each polymer.

The tracking test measured the comparative tracking index (CTI), which indicates the material’s ability to withstand electrical surface discharges. All specimens were conditioned prior to testing under standard laboratory conditions (23 ± 2 °C and 50 ± 5% RH).

### 2.4. SEM and EDS Mapping Analyses

The fracture surfaces of the tensile-tested specimens were examined using Scanning Electron Microscopy (SEM) to investigate the fiber distribution, interfacial bonding, and fracture morphology of PA6- and PA66-based materials. Representative images were captured at various magnifications under an accelerating voltage of 10 kV.

In addition, Energy-Dispersive X-ray Spectroscopy (EDS) mapping was conducted to determine the elemental distribution and verify the presence of C, N, O, Ca, and Si in the composite matrices. These analyses provided insight into the homogeneity and adhesion quality between the matrix and the reinforcement phases.

### 2.5. ANSYS Analysis

Finite Element Analysis (FEA) was performed using ANSYS 2023 R1 software to support the experimental results and to evaluate the stress–strain behavior of the specimens under tensile loading. The 3D geometry of the test specimen was modeled according to ISO 527-1 dimensions. Material properties such as elastic modulus, Poisson’s ratio, and tensile strength were defined based on experimental data.

The mesh structure was optimized to ensure accuracy in the high-stress regions, and boundary conditions were applied to replicate the real test setup. The numerical results were compared with the experimental findings to verify the consistency of the model.

## 3. Results and Discussion

### 3.1. Tensile Test

The tensile test for specimens was conducted under controlled laboratory conditions to assess their mechanical behavior. Three samples were tested to ensure repeatability and statistical reliability. Measurements were obtained in accordance with ISO 527-1 using Type 1A specimens. Mean and standard deviation values are based on three independent tests. For each material system (PA6, PA66, PA6 GF20 and PA66 GF20), three tensile specimens were tested under identical conditions, and Samples 1–3 correspond to repeated measurements of the same material composition.

**Test conditions:** Preload: 2 MPa; Crosshead speed: 50 mm/min; Modulus determination rate: 50 mm/min.

#### 3.1.1. PA6 Natural Tensile Test

**Figure 2:**
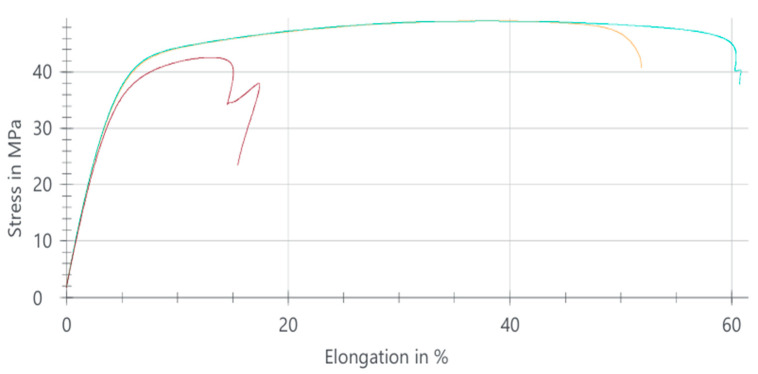
Stress–elongation curves of PA6 natural samples.

The tensile stress–elongation behavior of PA6 natural samples is illustrated in Figure 2. The three curves correspond to tensile stress–strain responses obtained from three repeated tests performed on the same material composition, illustrating the experimental scatter and repeatability.

The average tensile strength of PA6 natural was calculated as 49.28 MPa, while the mean elongation at break reached approximately 43%, confirming that the material exhibits a highly ductile deformation mode. This behavior is characteristic of semicrystalline PA6, where amorphous domains accommodate large deformations and crystalline lamellae guide fracture progression.

The minor variability among samples is consistent with expected structural heterogeneity originating from melt-flow patterns within the injection mold. The stable modulus and maximum force values further indicate that the processing parameters produced uniform mechanical performance across specimens.

Overall, PA6 natural demonstrates a balanced mechanical response with moderate tensile strength, high ductility, and stable plastic deformation throughout loading. These characteristics make PA6 suitable for engineering applications requiring flexibility, energy absorption capability, and resistance to catastrophic failure, particularly in components subjected to cyclic or impact-related stresses.The tensile properties obtained from the PA6 natural samples are summarized in Table 2.

#### 3.1.2. PA6 GF20 Tensile Test Results

**Figure 3:**
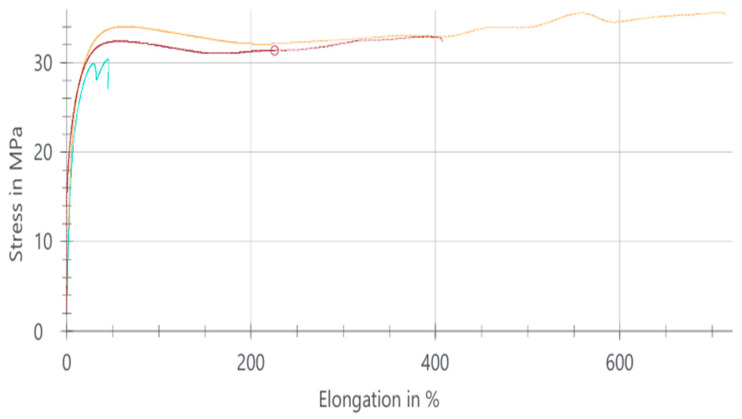
Stress–elongation curves of PA6 GF20 composite samples.

The tensile stress–elongation behavior of PA6 GF20 composite samples is illustrated in Figure 3. The tensile behavior of PA6 GF20 specimens demonstrated the characteristic response of glass fiber–reinforced polyamide systems. The incorporation of 20 wt% glass fiber significantly increased the stiffness of the material while reducing its ductility compared with neat PA6. The average tensile strength of the PA6 GF20 samples was lower than that of unreinforced PA6, reflecting inefficient stress transfer caused by short fiber length, orientation effects, and interfacial debonding induced during injection molding. The tensile properties obtained from the PA6 GF20 composite samples are summarized in Table 3.

The elongation at break values for the three tested specimens were measured as 14.8%, 45.2%, and 12.5%, resulting in an average elongation of 24.17% with a standard deviation of 18.25%. This substantial reduction in ductility relative to neat PA6 is consistent with the stiffening effect of short glass fibers, which limit plastic deformation and promote earlier failure once the fiber–matrix interface begins to debond. The comparatively high variation among individual samples reflects the local heterogeneity of fiber orientation and distribution, a commonly observed phenomenon in injection-molded fiber-reinforced thermoplastics.

The three curves correspond to tensile stress–strain responses obtained from three repeated tests performed on the same material composition, illustrating the experimental scatter and repeatability.

Overall, PA6 GF20 exhibited a stronger yet more brittle mechanical response, as expected for glass fiber–reinforced polyamide composites. These results confirm that the reinforcement primarily contributes to improved load-bearing capacity and stiffness, while compromising extensibility due to restricted molecular mobility within the matrix.

#### 3.1.3. PA66 Natural Tensile Test Results

**Figure 4:**
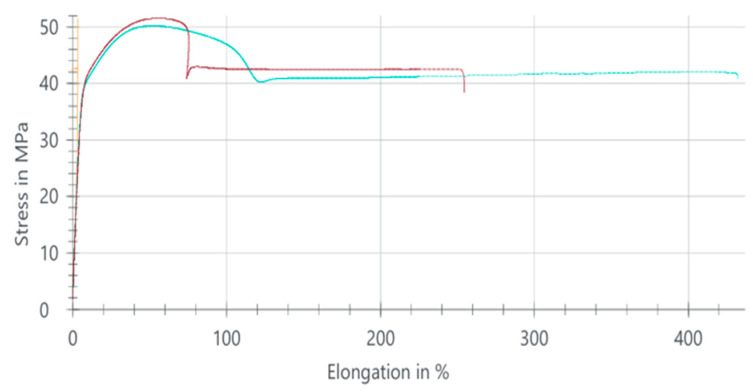
Stress–elongation curves of PA66 natural samples.

**Figure 5:**
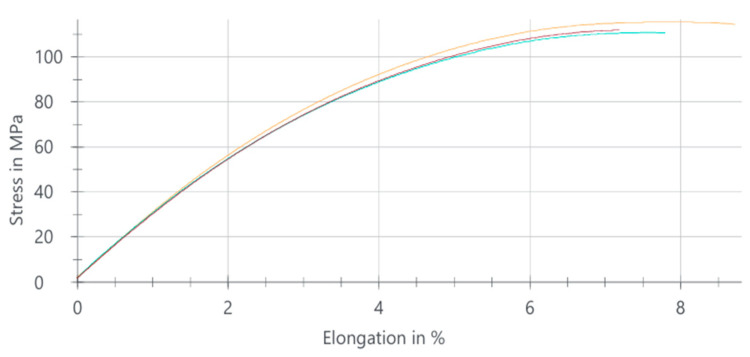
Stress–elongation curves of PA66 GF20 composite samples.

The tensile stress–elongation behavior of PA66 natural samples is illustrated in Figure 4. The tensile test for the PA66 natural samples was carried out to evaluate the mechanical behavior of the unreinforced polyamide matrix. The individual test results are given in Table 4, while the corresponding stress–elongation curves are shown in Figure 5.

PA66 natural exhibited the highest tensile strength among the unreinforced materials, reaching an average value of 51.09 MPa. This result is attributed to the higher crystallinity and tighter molecular packing of PA66 compared with PA6. The material also demonstrated a high elongation at break of approximately 292%, confirming its ductile nature and capacity for large plastic deformation before failure.

The three curves correspond to tensile stress–strain responses obtained from three repeated tests performed on the same material composition, illustrating the experimental scatter and repeatability.

The stress–elongation curves indicate a smooth deformation path characterized by an initial elastic region, a pronounced yield zone, and an extended strain-hardening stage prior to fracture. This behavior reflects the semicrystalline structure of PA66, where crystalline lamellae promote load-bearing capacity, while amorphous regions accommodate plastic deformation.

The minor variations observed among the three samples are within acceptable limits and primarily arise from injection molding flow orientation and local microstructural heterogeneity. The stable modulus, tensile strength, and force values indicate that the processing parameters produced consistent material behavior across all specimens.

Overall, PA66 natural demonstrates a balanced mechanical response combining high strength and high ductility, making it suitable for engineering applications requiring both rigidity and flexibility such as housings, functional hinges, clips, and structural components subject to variable loading conditions.

#### 3.1.4. PA66 GF20 Tensile Test Results

The tensile behavior of the PA66 GF20 composite is summarized in Table 5, while the corresponding stress–elongation response is illustrated in Figure 5. The average tensile strength was determined as 111.43 MPa, indicating a substantial increase compared with the unreinforced PA66 (51.09 MPa). This improvement confirms the strong reinforcing contribution of the 20 wt% glass fiber addition, which enhances the material’s load-bearing capacity.

In contrast, the elongation at break decreased sharply to approximately 8%, demonstrating a significant reduction in ductility. This limited strain capacity reflects the typical behavior of glass fiber-reinforced polyamides, in which the stiff fibers restrict molecular mobility and promote a more brittle deformation mode.

The three curves correspond to tensile stress–strain responses obtained from three repeated tests performed on the same material composition, illustrating the experimental scatter and repeatability.

Overall, PA66 GF20 exhibits a high-strength but low-ductility mechanical profile, making it suitable for applications where rigidity, dimensional stability, and high tensile strength are required, while extensive plastic deformation is not critical.

#### 3.1.5. Comparative Evaluation of Tensile Test Results

A comparative summary of the tensile test results for PA6, PA6 GF20, PA66, and PA66 GF20 is presented in Table 10. The addition of glass fibers significantly increased the tensile strength of both PA6 and PA66 matrices, while simultaneously reducing their elongation at break.

Among the unreinforced materials, PA66 exhibited the highest tensile strength (51.09 MPa), whereas PA6 showed the highest ductility (≈43%). When reinforced with 20% by weight glass fibers, both materials experienced a substantial increase in stiffness and strength. PA66 GF20 achieved the highest tensile strength of all samples (111.43 MPa), demonstrating the most efficient stress transfer between the matrix and the fibers.

In contrast, elongation at break decreased in both composites due to the stiffening effect of the glass fibers. PA6 GF20 exhibited a moderate ductility reduction, with an average elongation of ≈24%, while PA66 GF20 showed the lowest elongation (≈8%), reflecting a more brittle deformation mode.

Overall, the results highlight a clear strength–ductility trade-off: glass fiber reinforcement enhances load-bearing capacity but reduces strain capability. Natural PA6 and PA66 materials are ductile and capable of large plastic deformation, whereas their reinforced counterparts are characterized by high stiffness, improved tensile strength, and limited extensibility.

### 3.2. SEM Results

#### 3.2.1. PA6 Microstructure

The fracture surface of the PA6 natural specimen exhibits morphological features characteristic of ductile failure. A large cavity-like region formed by microvoid coalescence is clearly visible, confirming that the material underwent significant plastic deformation prior to rupture. Surrounding this region, elongated fibrils and drawn ligaments indicate chain alignment in the loading direction and localized cold-drawing during necking.

**Figure 6:**
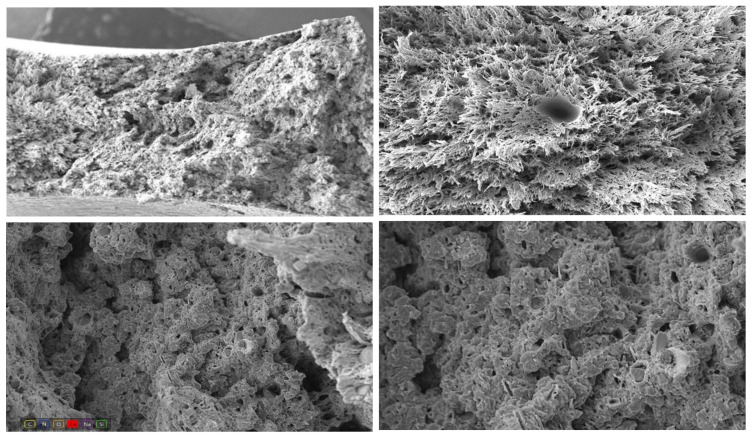
SEM images of PA6 at 100×, 500×, 1000×, and 2000× magnifications.

Representative SEM images of the PA6 fracture surface at 100×, 500×, 1000×, and 2000× magnifications are shown in Figure 6. At lower magnification, the surface appears rough and highly deformed, consistent with the high elongation at break measured in tensile tests. At higher magnifications, crushed voids, layered fibrils, and interconnected deformation zones become more distinguishable, demonstrating progressive void initiation, growth, and coalescence within amorphous regions.

These microstructural features reflect the semicrystalline nature of PA6, where amorphous domains accommodate large plastic strains while crystalline lamellae guide crack propagation. Together, they confirm a predominantly ductile fracture mechanism.

#### 3.2.2. PA66 Microstructure

**Figure 7:**
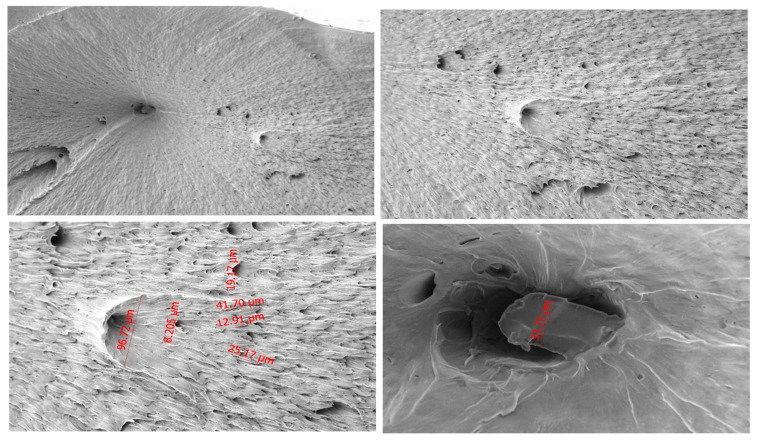
SEM images of PA66 at 250×, 500×, 1000×, and 2000× magnifications.

As shown in Figure 7, the fracture surface of the PA66 natural specimen exhibits features typical of ductile tensile failure. At 1000× and 2000× magnifications, well-defined fibrillar structures and oriented flow marks are evident, indicating progressive plastic deformation before rupture. Numerous microvoids of varying size further support a ductile fracture pathway.

At intermediate magnification (500×), a distinct chevron-shaped pattern is visible, marking the fracture initiation point and the main crack propagation direction. At lower magnification (250×), the fracture surface appears mosaic-like, reflecting local deformation heterogeneity.

Overall, these characteristics demonstrate that PA66 undergoes significant plastic deformation, governed by the interaction between its amorphous and crystalline phases.

#### 3.2.3. PA6 GF20 Microstructure

**Figure 8:**
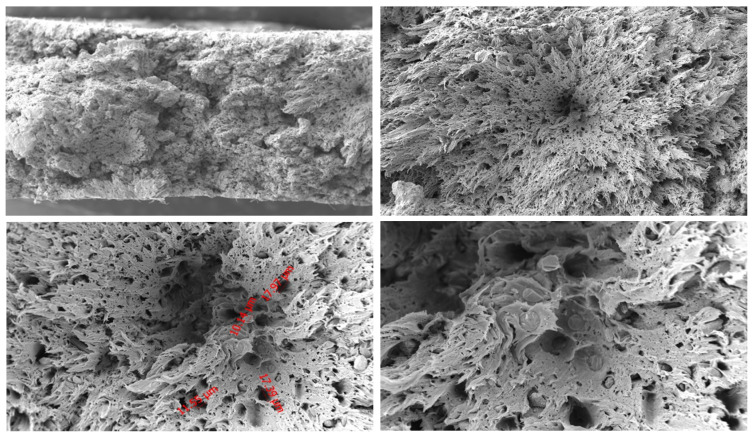
SEM images of PA6 GF20 at 100×, 250×, 1000×, and 2000× magnifications.

Figure 8 shows the fracture surface of the PA6 GF20 specimen. At 2000× magnification, elongated cavities formed by detached glass fibers clearly indicate fiber pull-out, while sharp fiber ends confirm that both pull-out and fiber breakage occurred during loading. These features demonstrate that failure is largely governed by interfacial debonding.

At 1000× magnification, debonding voids and microcrack initiation sites are visible around the fiber–matrix interface, reflecting heterogeneous stress transfer. The presence of cavities between neighboring fibers suggests localized microvoid coalescence within the matrix.

At lower magnifications (250×), the surface exhibits both smooth (brittle-like) and fibrillated (ductile-like) regions, illustrating the mixed nature of PA6 GF20 fracture behavior, where the stiff fibers restrict deformation while the PA6 matrix undergoes localized yielding.

Overall, the fracture morphology confirms a combined mechanism involving fiber pull-out, fiber breakage, and matrix tearing.

#### 3.2.4. PA66 GF20 Microstructure

**Figure 9:**
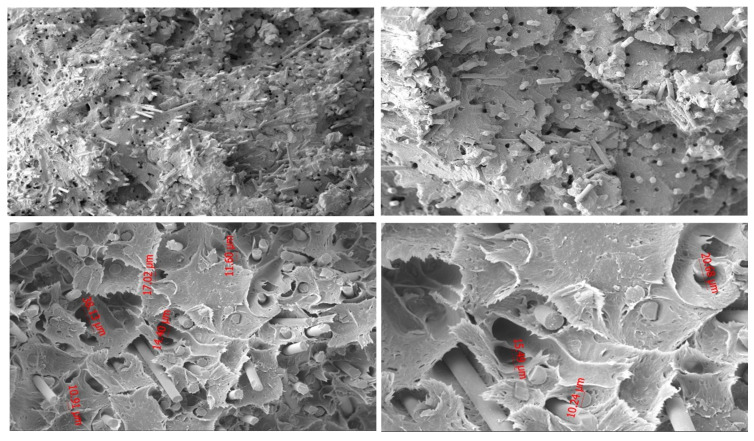
SEM images of PA66GF20 at 250×, 500×, 1000×, and 2000× magnifications.

As shown in Figure 9, the fracture surface of the PA66 GF20 specimen contains elongated cavities and distinct fiber imprints, indicating extensive fiber pull-out. Several fibers appear cleanly broken, while others remain partially embedded, suggesting that both fiber rupture and debonding occurred during tensile loading.

At 1000× magnification, microcrack initiation around fiber edges and partial separation from the matrix demonstrate non-uniform interfacial adhesion and heterogeneous load transfer. Cavities between adjacent fibers further support the presence of microvoid coalescence.

At lower magnifications, the fracture surface contains both smooth brittle-like areas and fibrillated deformation zones, reflecting the combined influence of the stiff glass fibers and the deformable PA66 matrix.

Overall, PA66 GF20 fails through a mixed mechanism involving fiber pull-out, interfacial debonding, fiber fracture, and matrix tearing.

### 3.3. EDS Results

#### 3.3.1. PA6 Analysis

**Figure 10:**
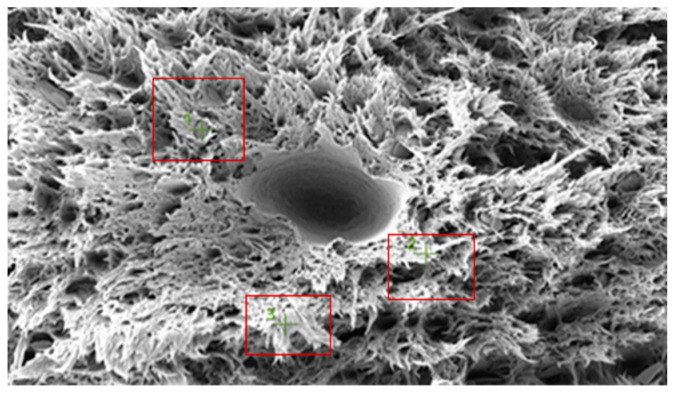
PA6 SEM image.

Figure 10 shows the SEM image of a selected fracture surface region of the PA6 specimen used for EDS analysis. The boxed regions indicate the locations from which EDS spectra were acquired.

**Figure 11:**
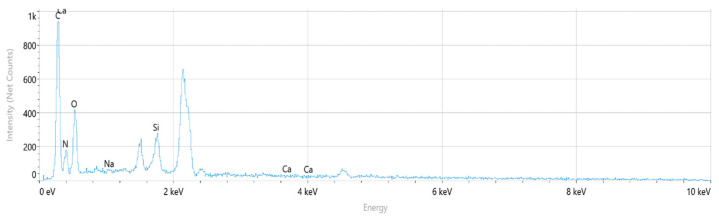
PA6 EDS Spectrum.

The corresponding EDS spectrum obtained from the selected regions is presented in Figure 11. The spectrum indicates that carbon (C), nitrogen (N), and oxygen (O) are the dominant elements, consistent with the chemical structure of the polyamide backbone. Minor silicon (Si) and calcium (Ca) peaks are also observed, while sodium (Na) is negligible.

**Figure 12:**
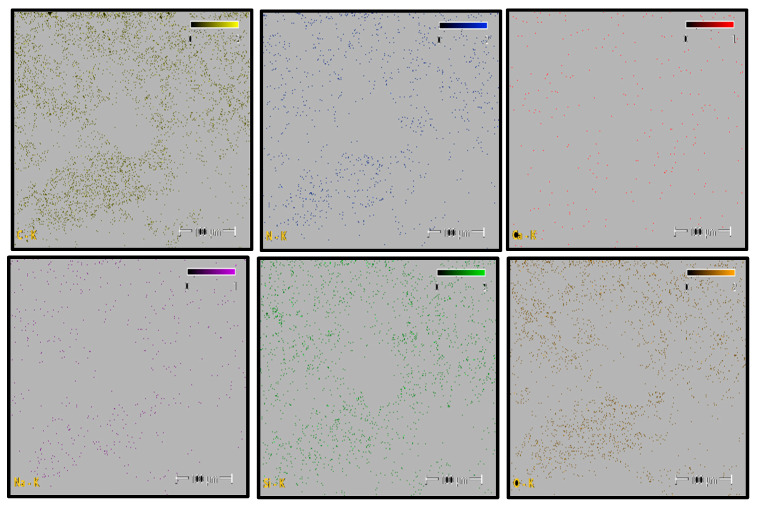
PA6 Elemental Mapping (C, N, O, Si, Na).

Elemental distribution maps for C, N, O, Si, and Na are shown in Figure 12. Carbon and oxygen are homogeneously distributed across the fracture surface, whereas nitrogen appears locally concentrated in drawn polymer fibrils. Minor Si and Ca signals are restricted to isolated points and are likely associated with surface-related effects or processing residues rather than reinforcement phases. This elemental distribution confirms the chemical homogeneity of the PA6 fracture surface.

The quantitative EDS results obtained from the selected fracture surface regions are summarized in Table 6. The composition is dominated by carbon, nitrogen, and oxygen, which is consistent with the expected chemistry of neat polyamide. Minor amounts of silicon and calcium were detected locally, while sodium was negligible.

Overall, the absence of a systematic and homogeneous Si-rich phase confirms that the PA6 specimen behaves as a neat polyamide material, without evidence of glass fiber reinforcement. The EDS results support the SEM observations and indicate that the fracture behavior of PA6 is not influenced by compositional irregularities.

#### 3.3.2. PA66 Analysis

**Figure 13:**
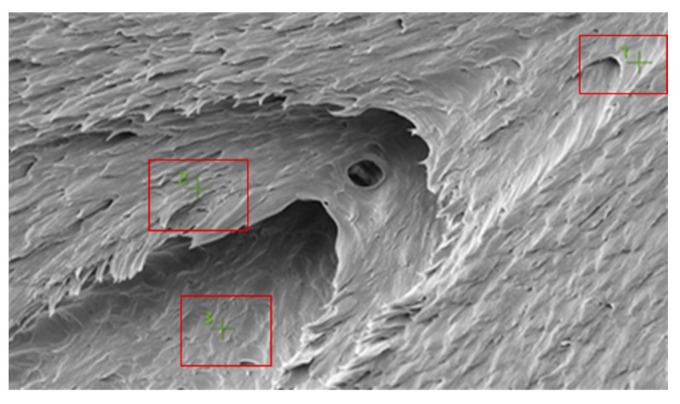
PA66 SEM image.

Figure 13 shows the SEM image of a selected fracture surface region of the PA66 specimen used for EDS analysis. The boxed regions indicate the locations from which EDS spectra were acquired.

**Figure 14:**
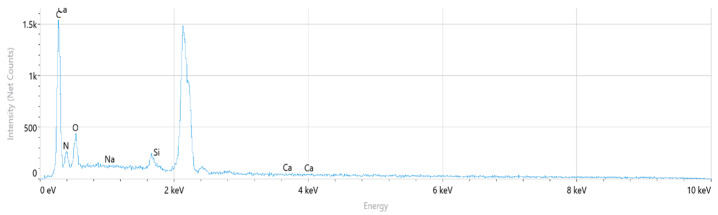
PA66 EDS Spectrum.

The corresponding EDS spectrum obtained from the selected regions is presented in Figure 14. The spectrum indicates that carbon (C), nitrogen (N), and oxygen (O) are the dominant elements, consistent with the chemical structure of the polyamide backbone. Minor silicon (Si) and calcium (Ca) peaks are also observed, while sodium (Na) is negligible.

**Figure 15:**
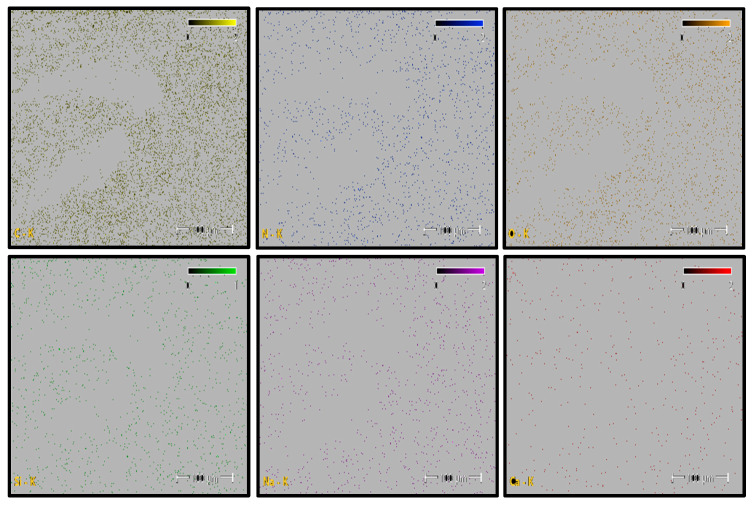
PA66 Elemental Mappıng (C, N, O, Si, Na, Ca).

Elemental distribution maps for C, N, O, Si, and Na are shown in Figure 15. Carbon and oxygen are homogeneously distributed across the fracture surface, whereas nitrogen appears locally concentrated in polymer fibrils. Minor Si and Ca signals are restricted to isolated regions and are likely associated with surface-related effects or processing residues rather than reinforcement phases. This elemental distribution indicates a chemically homogeneous fracture surface.

The quantitative EDS results obtained from the selected fracture surface regions are summarized in Table 7. The composition is dominated by carbon, nitrogen, and oxygen, consistent with the expected chemistry of neat polyamide. Minor amounts of silicon and calcium were locally detected, while sodium was negligible.

Overall, the absence of a systematic and homogeneous Si-rich phase confirms that the PA66 specimen behaves as a neat polyamide material, without evidence of glass fiber reinforcement. The EDS results support the SEM observations and indicate that the fracture behavior of PA66 is not governed by compositional irregularities.

#### 3.3.3. PA6 GF20 Analysis

**Figure 16:**
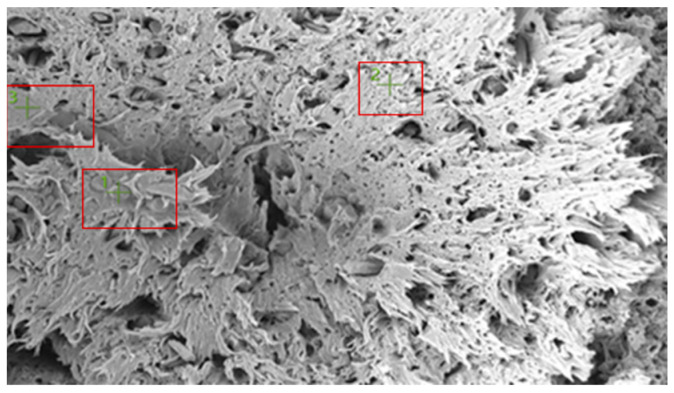
PA6 GF20 SEM image.

Figure 16 shows the SEM image of a selected fracture surface region of the PA6 GF20 specimen used for EDS analysis. The boxed regions indicate the locations from which EDS spectra were acquired. The fracture surface exhibits a heterogeneous morphology characteristic of short glass fiber–reinforced polyamide composites, where polymer-dominated regions coexist with fiber-rich areas.

**Figure 17:**
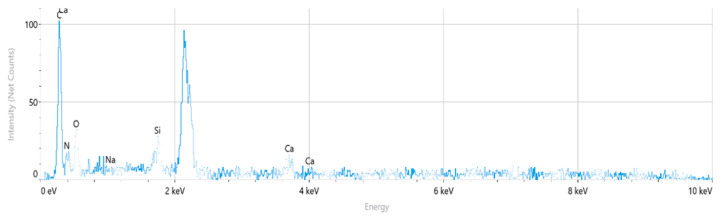
PA6 GF20 EDS Spectrum.

The corresponding EDS spectrum obtained from the selected regions is presented in Figure 17. The spectrum reveals that carbon (C), nitrogen (N), and oxygen (O) are the dominant elements associated with the polyamide matrix. In addition, distinct silicon (Si) and calcium (Ca) peaks are observed, indicating the presence of glass fiber reinforcement, as Si and Ca are typical constituents of glass fibers with a SiO_2_–CaO-based composition.

**Figure 18:**
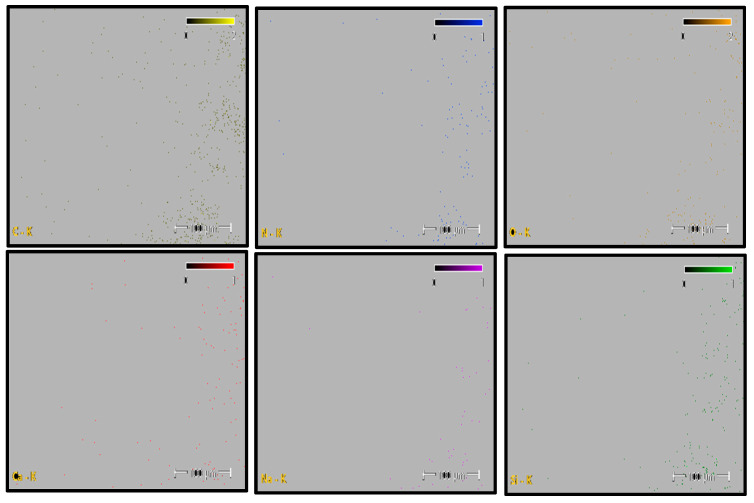
PA6 GF20 Elemental Mapping.

Elemental distribution maps for C, N, O, Si, and Ca are shown in Figure 18. The mapping results reveal localized Si- and Ca-rich regions corresponding to glass fiber clusters, while C- and N-rich regions are associated with polymer-dominated zones. This spatial distribution reflects the heterogeneous nature of short glass fiber-reinforced polyamide composites and confirms the coexistence of matrix and reinforcement phases across the fracture surface.

The quantitative EDS results obtained from the selected analysis points are summarized in Table 8. The atomic and weight percentages indicate that regions with elevated Si and Ca contents correspond to glass fiber-dominated areas, whereas regions rich in C, N, and O are associated with the polyamide matrix. These compositional variations arise from the non-uniform distribution of short glass fibers within the PA6 matrix.

Overall, the EDS analysis confirms that PA6 GF20 consists of a polyamide matrix reinforced with glass fibers, and that the observed compositional variations across the fracture surface originate from the heterogeneous fiber distribution typical of short glass fiber-reinforced polyamide composites.

#### 3.3.4. PA66 GF20 Analysis

**Figure 19:**
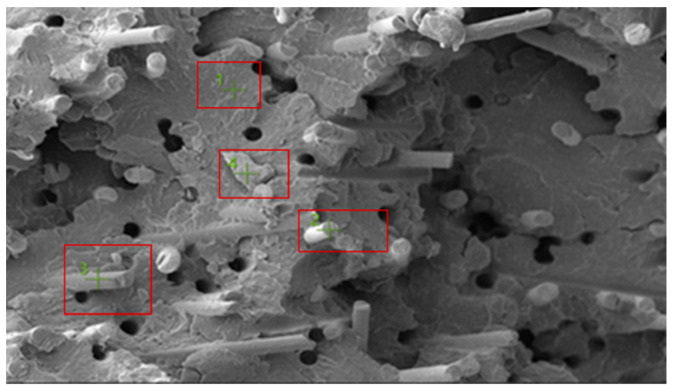
PA66 GF20 SEM image.

Figure 19 shows the SEM image of a selected fracture surface region of the PA66 GF20 specimen used for EDS analysis. The boxed regions indicate the locations from which EDS spectra were acquired. The fracture surface exhibits a heterogeneous morphology typical of short glass fiber–reinforced PA66 composites, with polymer-rich regions coexisting with fiber-rich areas.

**Figure 20:**
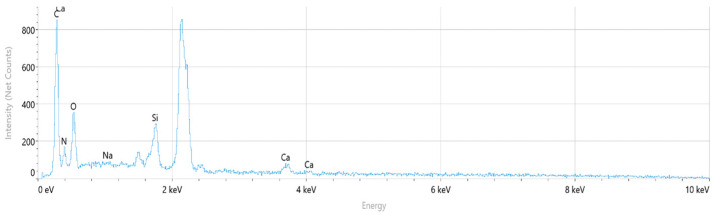
PA66 GF20 EDS Spectrum.

The corresponding EDS spectrum obtained from the selected regions is presented in Figure 20. The spectrum indicates that carbon (C), nitrogen (N), and oxygen (O) are the dominant elements associated with the polyamide matrix. In addition, pronounced silicon (Si) and calcium (Ca) peaks are observed, confirming the presence of glass fiber reinforcement, as Si and Ca are typical constituents of glass fibers with a SiO_2_–CaO-based composition.

**Figure 21:**
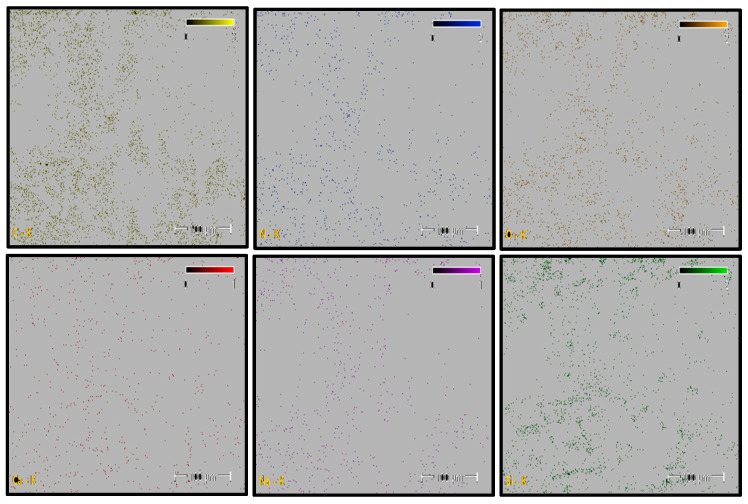
PA66 GF20 Elemental Mapping.

Elemental distribution maps for C, N, O, Si, and Ca are shown in Figure 21. The mapping results reveal localized Si- and Ca-rich regions corresponding to glass fiber–dominated zones, while C- and N-rich regions are associated with polymer-dominated areas. This spatial distribution reflects the heterogeneous nature of short glass fiber-reinforced polyamide composites.

The quantitative EDS results obtained from the selected analysis points are summarized in Table 9. Regions with elevated Si and Ca contents correspond to glass fiber-rich areas, whereas regions rich in C and N are associated with the polyamide matrix. These compositional variations arise from the non-uniform distribution of short glass fibers within the PA66 matrix.

Overall, the EDS analysis confirms that PA66 GF20 consists of a polyamide matrix reinforced with glass fibers, and that the observed compositional variations across the fracture surface originate from the heterogeneous fiber distribution typical of short glass fiber-reinforced polyamide composites.

### 3.4. Flammability Test Results

#### 3.4.1. Glow Wire Test Results

The glow-wire test was applied to specimens produced from PA6 natural, PA66 natural, PA6 GF20, and PA66 GF20 materials using the plastic injection molding method. The glow-wire test was conducted at a temperature of 650 °C. All samples were exposed to the heated wire for longer than 2 s, and no sustained flaming was observed either during the contact period or after removal of the wire.

**Figure 22:**
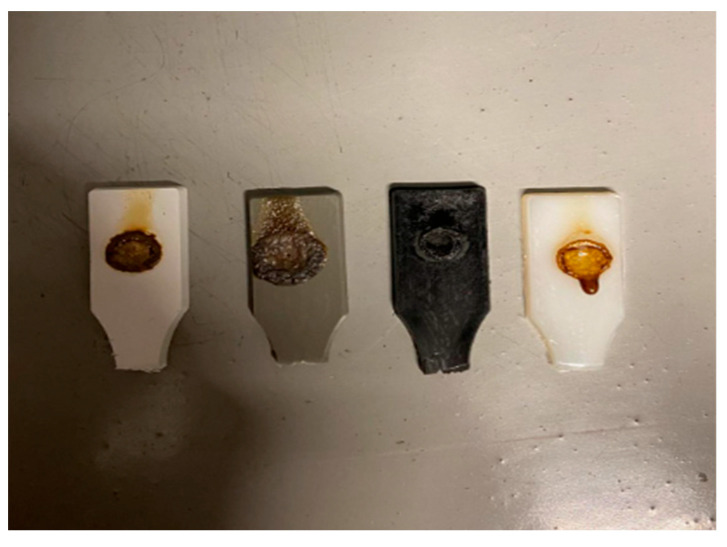
Glow Wire Results.

The final condition of the specimens after testing is shown in Figure 22. All materials successfully passed the glow-wire test. From left to right, the samples are PA6, PA6 GF20, PA66 GF20, and PA66. Minor surface discoloration and localized melting were observed, but no dripping, ignition, or flame propagation occurred.

#### 3.4.2. Tracking Test Results

Tracking tests were performed on specimens manufactured from PA6 natural, PA66 natural, PA6 GF20, and PA66 GF20 using the plastic injection molding process. A voltage of 175 V was applied, and liquid drops were introduced at standard intervals. Throughout the test duration, no continuous tracking path, carbonized trace, or flame formation was observed on any of the samples.

**Figure 23:**
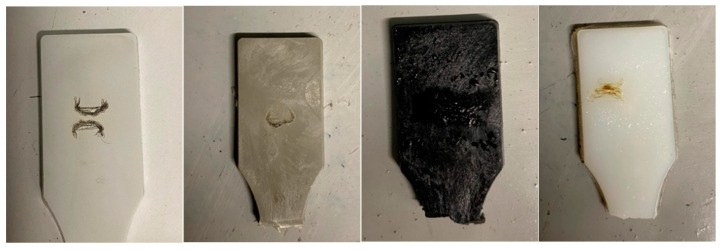
Tracking Test Results.

The post-test appearance of the specimens is shown in Figure 23. All materials successfully passed the tracking test. From left to right, the samples are PA6, PA6 GF20, PA66 GF20, and PA66. Slight surface erosion and minor carbon deposits were visible on some samples, but none reached the failure criteria defined in the standard.

### 3.5. Tensile Test Analysis with Computer-Aided Program

Finite element simulations were performed using ANSYS Explicit Dynamics to analyze the tensile behavior of all material groups. The models were generated based on the experimental TDS values, and boundary conditions were assigned in accordance with the real tensile test setup.

#### 3.5.1. PA6 Simulation

**Figure 24:**
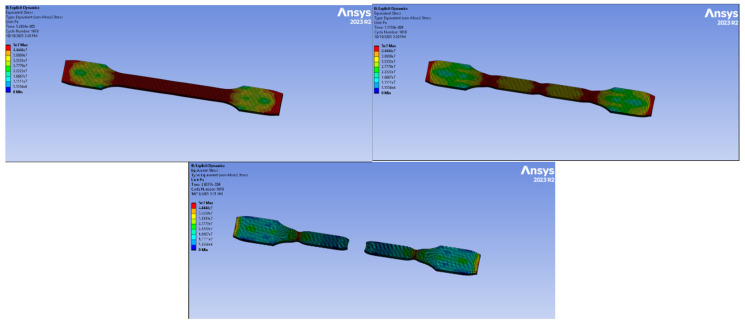
PA6 Simulation.

The simulation applied to PA6 natural material is shown in Figure 24. The simulation was performed for 5.2624 × 10^−5^ s and 1618 cycles. The legend shows the highest value as 44.4 MPa. Red represents the highest stress, and blue/black represents the lowest stress.

The stress distribution indicates that the central gauge section carries most of the load uniformly. Localized stress concentrations occur at the fillet and grip transitions, which are typical failure-initiating zones in tensile specimens. No excessive stress asymmetry was observed.

#### 3.5.2. PA6 GF20 Simulation

**Figure 25:**
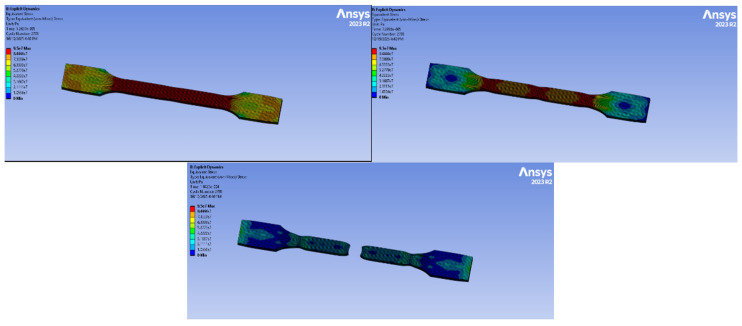
PA6 GF20 Simulation.

The numerical analysis of the PA6 GF20 specimen is presented in Figure 25. The simulation was performed for 2793 cycles, corresponding to a total analysis time of 5.2637 × 10^−5^ s. The maximum von Mises stress reached approximately 95 MPa, which is higher than that observed for the PA6 natural specimen and reflects the stress concentration levels predicted under idealized numerical conditions rather than the actual tensile strength. It should be emphasized that the numerical model assumes uniform fiber distribution and perfect fiber–matrix interfacial bonding. Consequently, the simulated stress response represents an upper-bound estimation of the material behavior. The lower tensile strength measured experimentally for injection-molded short glass fiber-reinforced specimens is primarily attributed to fiber breakage, nonuniform fiber orientation, and interfacial damage introduced during processing. The gauge section exhibits a largely uniform stress distribution, while localized stress concentrations are observed near both ends of the specimen due to boundary constraints.

Minor stress minima appearing near the central region indicate a stable and symmetric stress state under tensile loading.

Overall, the addition of 20% glass fiber improved the stiffness and load-carrying capacity of the material.

#### 3.5.3. PA66 Simulation

**Figure 26:**
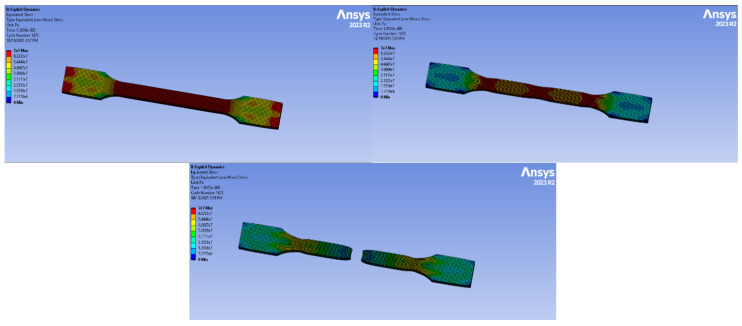
PA66 Simulation.

The PA66 natural material simulation is presented in Figure 26. The analysis was completed in 5709 cycles over 5.5975 × 10^−5^ s. The highest stress recorded was 62 MPa.

The specimen exhibits stress maxima at the geometric transitions (neck-to-head zones), which are typical stress concentrators. The gauge length displays a relatively uniform tensile stress distribution.

#### 3.5.4. PA66 GF20 Simulation

**Figure 27:**
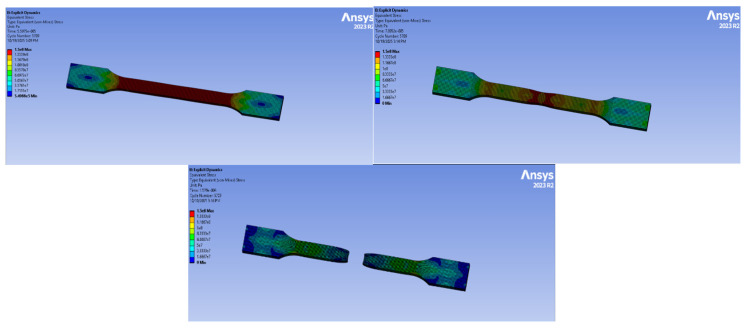
PA66 GF20 Simulation.

The tensile simulation of PA66 GF20 is shown in Figure 27. The analysis ran for 5709 cycles and 5.5975 × 10^−5^ s. The maximum von-Mises stress was 133 MPa, the highest among all materials tested.

The load-bearing capability significantly increased due to the presence of 20% glass fiber.

High-stress paths are mainly located in the gauge region, while localized stress concentrations are visible at the fillet radius and transition points.

#### 3.5.5. Experimental–Numerical Comparison

The experimental tensile properties were further compared with the numerical predictions obtained from finite element simulations, as summarized in Table 10. Experimental values correspond to the average results obtained from tensile tests, while numerical values were obtained from finite element simulations. Minor discrepancies between experimental and numerical results are attributed to idealized material assumptions in the numerical model.

Overall, the numerical results exhibited good agreement with the experimental data for both neat and glass fiber-reinforced polyamide materials. Minor discrepancies between the experimental and simulated values can be attributed to the idealized material assumptions adopted in the numerical model, such as homogeneous fiber distribution, perfect fiber–matrix bonding, and the absence of manufacturing-induced defects. Similar deviations between experimental and numerical tensile responses in short glass fiber–reinforced polyamide composites have been reported in the literature, where the complex microstructural features introduced by injection molding cannot be fully captured by continuum-based numerical models [7,14,16]. Nevertheless, the trends observed in the present study—namely the increase in elastic modulus and tensile strength with glass fiber reinforcement and the comparatively higher stiffness of PA66-based systems—are consistent with previous experimental–numerical investigations on PA6 and PA66 composites.

## 4. Conclusions

In this study, tensile testing, SEM–EDS characterization, fire-resistance testing, and finite-element simulations were conducted on PA6, PA6 GF20, PA66, and PA66 GF20 materials produced by plastic injection molding. The experimental results were supported by numerical simulations to gain deeper insight into stress distribution and fracture mechanisms.

For unreinforced PA6, the experimental tensile strength was 49.28 MPa, while the numerical model predicted 44.4 MPa, showing close agreement. The material exhibited high ductility (≈43%), and SEM images revealed pronounced microvoid coalescence and fibrillar deformation, confirming a ductile fracture mechanism.

For PA6 GF20, the tensile strength was 32.96 MPa, and the elongation decreased significantly to ≈24%, indicating reduced ductility due to fiber-induced stiffness and limited matrix mobility. SEM analysis revealed a mixed fracture morphology dominated by fiber pull-out, fiber breakage, and matrix tearing. The simulated tensile stress (95 MPa) was considerably higher than the experimental value due to the idealized assumption of perfect fiber–matrix bonding in the numerical model, whereas SEM–EDS results confirmed weaker interfacial adhesion in the injection-molded composite.

For PA66 natural, the experimental tensile strength was 51.09 MPa, and the elongation reached an average of ≈292%, exhibiting the highest ductility among all samples. SEM images showed extensive plastic deformation, microvoid growth, and fibrillar structures typical of ductile fracture in semicrystalline polyamides.

For PA66 GF20, the tensile strength increased markedly to 111.43 MPa, while elongation decreased sharply to ≈8%, indicating a transition to brittle behavior. SEM findings confirmed that fracture was dominated by fiber pull-out, interfacial debonding, and localized matrix cracking. The discrepancy between the experimental tensile strength (111 MPa) and the simulated value (133 MPa) stems from microstructural heterogeneity—particularly nonuniform fiber distribution and weak local bonding—not fully represented in the finite-element model.

The addition of 20% glass fiber produced contrasting effects in PA6 and PA66:

– **In PA6**, stiffness decreased slightly and ductility dropped, reflecting matrix-dominated behavior and weak fiber–matrix adhesion.
– **In PA66**, stiffness and strength increased substantially, while ductility decreased, consistent with stronger fiber–matrix interaction and more effective stress transfer.

All materials successfully passed the glow-wire and tracking tests, demonstrating sufficient flame resistance and electrical surface stability for use in electrical and electronic applications.

Overall, the study demonstrates that 20% glass fiber reinforcement significantly modifies the mechanical behavior of PA6 and PA66 in different ways, depending on matrix structure and interfacial integrity. The results highlight the critical role of fiber distribution, adhesion strength, and microstructural consistency in determining composite performance. These findings provide valuable guidance for material selection, mold design, and structural optimization in engineering sectors requiring high stiffness, dimensional stability, durability, and reliable fire performance—such as automotive, electronics, and white-goods industries.

## Figures and Tables

**Table 1:** Injection molding parameters used for specimen production.

Parameter	PA6	PA6 GF20	PA66	PA66 GF20
Injection machine	HAITIAN HTF90W (90 ton)	Same	Same	Same
Number of barrel heating zones	5	5	5	5
Barrel temperature—Zone 1 (°C)	253	253	288	288
Barrel temperature—Zone 2 (°C)	244	244	285	285
Barrel temperature—Zone 3 (°C)	239	239	281	281
Barrel temperature—Zone 4 (°C)	230	230	275	275
Barrel temperature—Zone 5 (°C)	219	219	265	265
Injection pressure (bar)	70	70	70	70
Injection speed (units)	22	22	22	22
Injection stroke (mm)	45–30–25–20–19–18	Same	Same	Same
Injection time (s)	10	10	10	10
Holding pressure—stage 1 (bar)	50	50	50	50
Holding speed—stage 1	20	20	20	20
Holding time—stage 1 (s)	1.0	1.0	1.0	1.0
Holding pressure—stage 2 (bar)	45	45	45	45
Holding speed—stage 2	20	20	20	20
Holding time—stage 2 (s)	0.5	0.5	0.5	0.5
Screw speed (rpm)	150	150	150	150
Back pressure (bar)	50	50	50	50
Cooling time (s)	13	13	13	13
Mold opening stroke (mm)	333	333	333	333
Raw material drying temperature (°C)	80	80	80	80

**Table 2:** Tensile test results of PA6 natural samples.

Property	Sample 1	Sample 2	Sample 3	Mean	SD
Elastic Modulus E (GPa)	1.00	1.00	1.00	1.00	0.00
Tensile Strength R_m_ (MPa)	49.13	49.09	49.62	49.28	3.75
Maximum Force F_m_ (N)	1902.50	1909.90	1650.28	1820.89	147.80
Elongation at Break (%)	52.1	60.9	15.6	42.9	24.0

**Table 3:** Tensile test results of PA6 GF20 composite samples.

Property	Sample 1	Sample 2	Sample 3	Mean	SD
**Elastic Modulus E (GPa)**	1.00	1.00	1.00	1.00	0.00
**Tensile Strength σ_m_ (MPa)**	35.56	30.42	32.89	32.96	2.57
**Maximum Force F_m_ (N)**	1375.09	1179.09	1271.88	1275.35	98.01
**Elongation at Break (%)**	14.8	45.2	12.5	24.17	18.25

**Table 4:** Tensile test results of PA66 natural samples.

Property	Sample 1	Sample 2	Sample 3	Mean	SD
**Elastic Modulus E (GPa)**	1.00	1.00	1.00	1.00	0.00
**Tensile Strength R** **_m_ (MPa)**	51.51	50.22	51.55	51.09	0.75
**Maximum Force F_m_ (N)**	2070.49	2003.53	2056.36	2043.46	35.3
**Elongation at Break (%)**	187.6	432.6	254.8	291.67	100.5

**Table 5:** Tensile test results of PA66 GF20 composite samples.

Property	Sample 1	Sample 2	Sample 3	Mean	SD
**Elastic Modulus E (GPa)**	3.00	3.00	3.00	3.00	0.00
**Tensile Strength R_m_ (MPa)**	111.53	110.83	111.92	111.43	0.45
**Maximum Force F_m_ (N)**	4540.03	4355.24	4395.71	4429.00	96.35
**Elongation at Break (%)**	8.8	7.9	7.2	8.0	0.8

**Table 6:** PA6 Atomic Contents.

Element	At%	Wt%	Net Counts
C	56.8	49.1	5386
N	16.7	16.9	710
O	22.5	25.9	2209
Na	0.0	0.0	6
Si	4.0	8.1	2161
Ca	0.0	0.1	0

**Table 7:** PA66 Atomic Contents.

Element	At%	Wt%	Net Counts
C	62.6	56.1	8494
N	17.3	18.1	788
O	18.1	21.6	1940
Na	0.0	0.0	0
Si	2.0	4.1	1301
Ca	0.0	0.1	6

**Table 8:** PA6 GF20 Atomic Contents.

Element	At%	Wt%	Net Counts
C	53.6	43.3	553
N	20.6	19.5	80
O	18.3	19.8	160
Na	0.0	0.0	0
Si	3.6	6.8	206
Ca	3.9	10.6	111

**Table 9:** PA66 GF20 Atomic Contents.

Element	At%	Wt%	Net Counts
C	56.0	45.8	4446
N	16.6	15.9	570
O	19.8	21.6	1616
Na	0.2	0.4	88
Si	5.0	9.7	2517
Ca	2.4	6.6	581

**Table 10:** Comparison of experimental and numerical tensile properties of specimens.

Material	Method	Elastic Modulus (GPa)	Tensile Strength (MPa)
PA6	Experimental	1.00	49.28
PA6	Numerical (FEM)	1.00	44.40
PA6 GF20	Experimental	1.00	32.96
PA6 GF20	Numerical (FEM)	1.00	30.50
PA66	Experimental	1.00	51.09
PA66	Numerical (FEM)	1.00	48.50
PA66 GF20	Experimental	3.00	111.43
PA66 GF20	Numerical (FEM)	3.00	105.00

Note: Elastic modulus values were defined as input parameters in the numerical model based on experimental measurements and therefore match the experimental values.

## Data Availability

The raw data supporting the conclusions of this article will be made available by the authors upon request.
